# Supplementary material for: Mycorrhizal C/N ratio determines plant-derived carbon and nitrogen allocation to symbiosis
Source: Commun Biol. 2023 Dec 5;6:1230. doi: 10.1038/s42003-023-05591-7 (PMC10698078; doi:10.1038/s42003-023-05591-7)
Supplement: Supplementary file 2 — Supplementary Information [file 42003_2023_5591_MOESM2_ESM.pdf]

Supplementary Information

Mycorrhizal C/N ratio determines plant-derived carbon and nitrogen allocation to symbiosis

Rodica Pena[1,2], Sarah L. Bluhm[3], Silke Ammerschubert[1], Paola Agüi-Gonzalez[4], Silvio O. Rizzoli[4], Stefan Scheu[3,5] and Andrea Polle[1,5]*

[1] Forest Botany and Tree Physiology, University of Göttingen, Göttingen, Germany.

[2] Department of Sustainable Land Management and Soil Research Centre, School of Agriculture Policy and Development, University of Reading, Reading, United Kingdom.

[3] J.F. Blumenbach Institute of Zoology and Anthropology, Animal Ecology, University of Göttingen, Göttingen, Germany.

[4] Department of Neuro- and Sensory Physiology and Center for Biostructural Imaging of Neurodegeneration, University Medical Center Göttingen, Germany.

[5] Centre for Biodiversity and Sustainable Land Use, University of Göttingen, Göttingen, Germany.

Contents

Supplementary Table S1. Estimated fractions of fungal structures and their biomass within ectomycorrhizas.

Supplementary Table S2. Analysis of variance for ^{13}C, C, ^{15}N and N in EM species at two-time points after labeling.

Supplementary Figure S1. Experimental set-up for $^{13}CO_2$ exposure of trees.

Supplementary Figure S2. Typical collection scheme for ectomycorrhizas, the attached lateral root segment and a cross section of a typical ectomycorrhiza.

Supplementary Figure S3. Amounts of newly acquired ^{13}C and ^{15}N in fungal biomass per plant and in relation to the C/N ratio of the root tip.

Supplementary Figure S4. SIMS (Secondary Ion Mass Spectrometry) imaging of ^{14}N and ^{15}N in labelled and non-labelled ectomycorrhizas with the fungus *Pachyphlodes conglomerata*.

Supplementary Figure S5. Relationships between ^{13}C enrichment and ^{15}N enrichment with the C and the N concentrations in different EM fungal species.

Supplementary Tables

Supplementary Table S1. Estimated fractions of fungal structures and their biomass within ectomycorrhizas.

Ectomycorrhiza	Fungal tissue (%)	Fungal biomass (µg)
Pc	37.50 ± 1.93	2.9
UEM1	26.05 ± 0.59	2.4
UEM2	31.09 ± 1.14	2.9
UEM3	25.33 ± 0.38	2.4
Tp	49.95 ± 2.79	5.2
Cg	31.30 ± 1.34	1.8
To_1	37.78 ± 0.05	3.9

The relative fraction of fungal structures in a given ectomycorrhiza was determined by measuring the total area of a cross section and the area occupied by fungal tissue in the same cross section as:

Fungal tissue (%) = area occupied by the fungal tissue x 100 / total cross sectional area. Ectomycorrhizal fungal species names are abbreviated as follows: Pc: *Pachyphlodes conglomeratae* (n = 9), UEM1: uncultured EM 1 (n = 3), UEM2: uncultured EM 2 (n = 6), UEM3: uncultured EM 3 (n = 2), Tp: *Tomentella punicea* (n = 9), Cg: *Cenococcum geophilum* (n = 9), To1: Tomentella sp1 (n = 2). Number n in brackets indicates the number of cross-sections obtained from individual trees. The values represent means (± SE). The mean value of the root tip weight of distinct ectomycorrhizal species was retrieved from [1,2]. Fungal biomass of given ectomycorrhizal root tips was calculated as:

Fungal biomass (µg) = biomass of EM root tip x fungal tissue /100

Supplementary Table S2. Analysis of variance for ^{13}C, C, ^{15}N and N in EM species at day 5 and day 20 after labeling.

Source	Sum of Squares	Df	Mean Square	F-Ratio	P-Value
Main effects ---------------------------- ^{13}C in EM (mg g^{-1} DM) ----------------------------					
EM species	4701.9	6	783.6	7.8	<0.001
Time	1.8	1	1.8	0.0	0.894
Interactions					
EM species x time	310.6	6	51.8	0.5	0.791
Residual	2315.2	23	100.7		
Total(corrected)	7592.5	36			
Main effects ----------------------------- C in EM (mg g^{-1} DM) -----------------------------					
EM species	15284.2	6	2547.4	3.2	0.020
Time	35.4	1	35.4	0.0	0.835
Interactions					
EM species x time	3454.1	6	575.7	0.7	0.634
Residual	18271.3	23	794.4		
Total(corrected)	38686.3	36			
Main effects ----------------------------^{15}N in EM (µg g^{-1} DM) ------------------------------					
EM species	689.0	6	114.8	1.6	0.188
Time	41.6	1	41.6	0.6	0.452
Interactions					
EM species x time	27.1	6	4.5	0.1	0.999
Residual	1636.2	23	71.1		
Total(corrected)	2502.8	36			
Main effects ---------------------------- N in EM (mg g^{-1} DM) ------------------------------					
EM species	1474.2	6	245.7	10.0	<0.001
Time	31.3	1	31.3	1.3	0.267
Interactions					
EM species x time	34.2	6	5.7	0.2	0.961
Residual	563.3	23	24.5		
Total(corrected)	2144.8	36			

Supplementary Figure S1. Experimental set-up for $^{13}CO_2$ exposure of trees

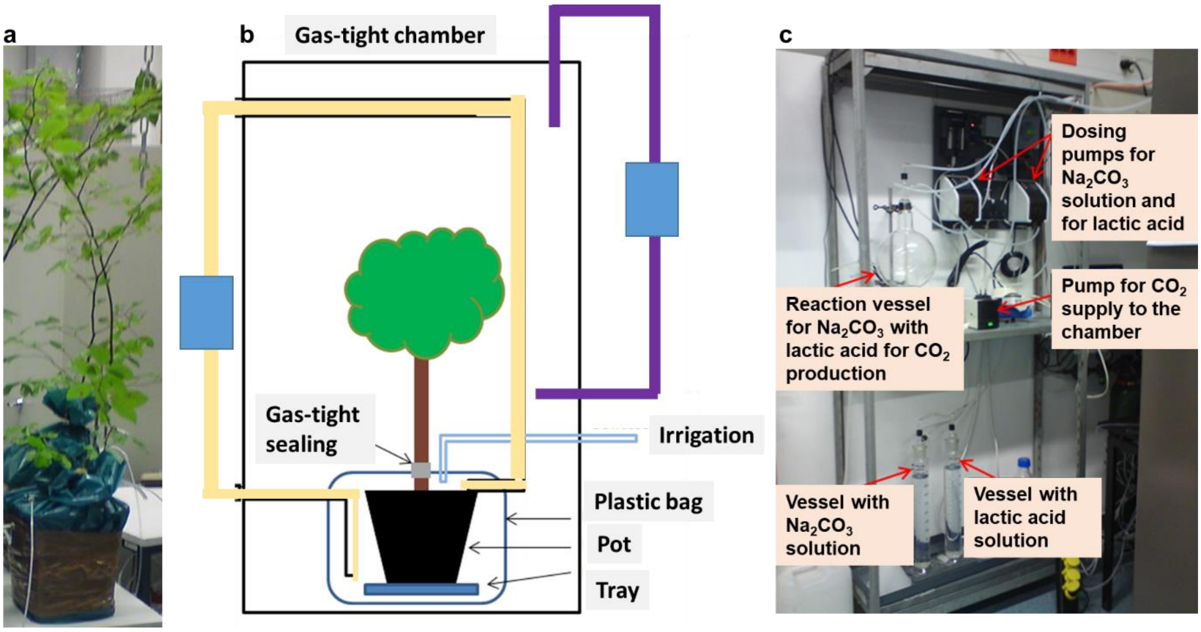

(a) Young beech tree with the pot enwrapped in plastic bag. Tubings for aeration (yellow arrow) and for irrigation (blue arrow) are indicated. Pot height is 0.25 m. **(b)** Scheme of the gas-tight chamber with a plant and the separation of the aeration system of the belowground compartment (yellow) and the aboveground compartment (purple). Blue squares symbolize stations for pumping, measuring and mixing systems. **(c)** Station for the production and supply with $^{13}CO_2$. The original schemes were provided by S. Bluhm and modified.

The reaction solutions (106.98 g $Na_2{}^{13}CO_3$ [containing 99% ^{13}C, Sigma Aldrich, Taufkirchen, Deutschland] in 2 L distilled water and 5M solution of lactic acid [$C_3H_6O_3$ 90% pur Ph Eur. USP, AppliChem GmbH, Darmstadt, Deutschland] are stored in two separate vessels. When the target concentration of CO_2 in the aboveground aeration system dropped below the pre-set threshold (here 180 ppm CO_2), the dosing pumps were activated and pumped the solutions into a reaction vessel, where CO_2 was released by the following reaction:

$$Na_2{}^{13}CO_3 + 2\ C_3H_6O_3 \text{ -> } 2\ NaC_3H_5O_3 + {}^{13}CO_2 + H_2O$$

CO_2 was then supplied by a pump to the aboveground aeration system. Thereby, the CO_2 concentration increased and the pumps stopped when the upper threshold of 400 ppm was reached.

Supplementary Figure S2. Typical collection scheme for ectomycorrhizas, the attached lateral root segment and a cross section of a typical ectomycorrhiza.

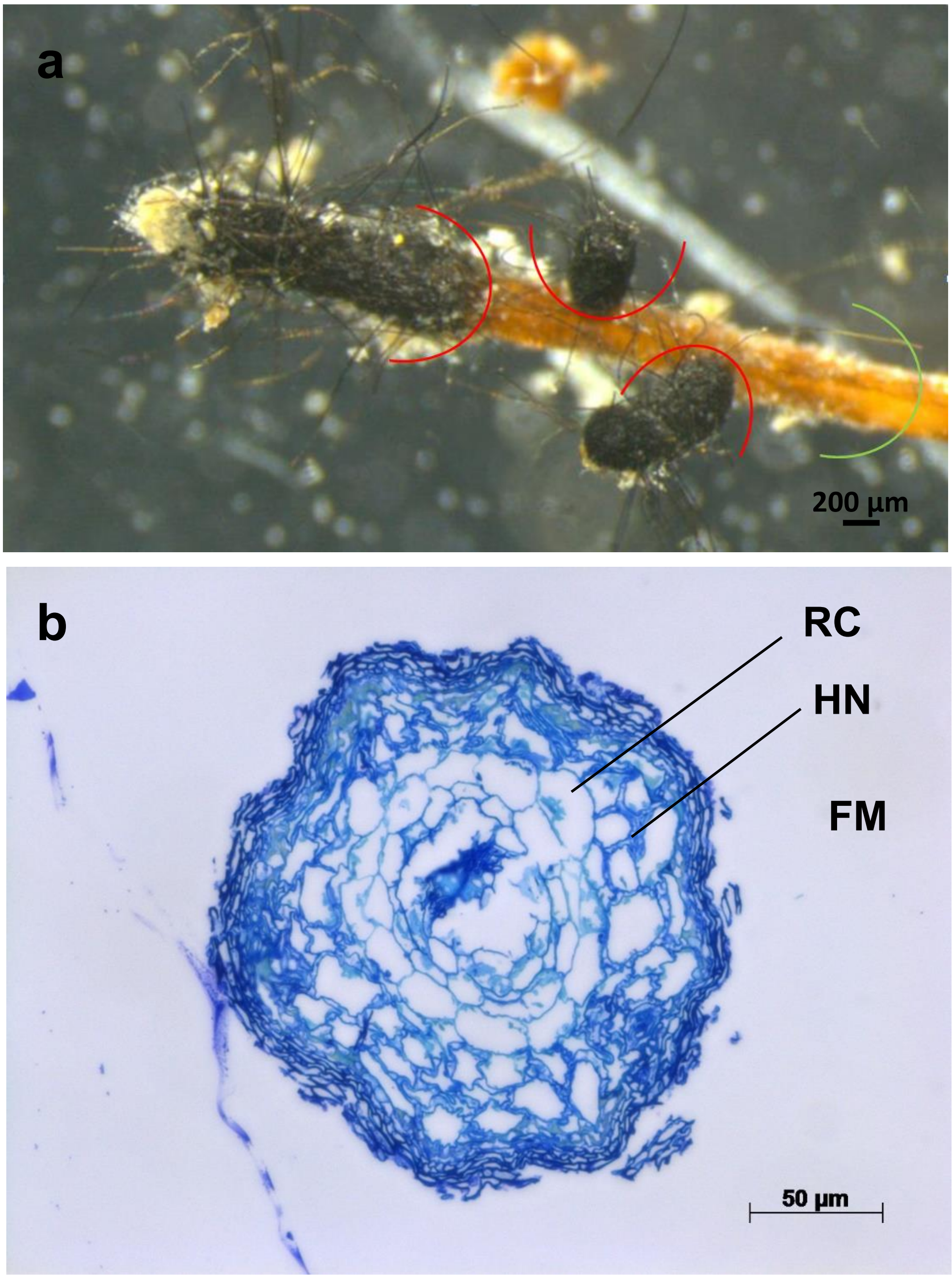

(a) Typical collection scheme for ectomycorrhizas (cut as indicated by the red line) and the attached lateral root segment (cut as indicated by the green line). The picture shows *Cenococcum geophilum*; **(b)** Cross section of a typical ectomycorrhiza with an uncultured ectomycorrhizal fungus (UEM) belonging to the Helotiales. The picture shows UEM 2 (Helotiales). RC = root cortex cell, HN = Hartig net, FM = Fungal mantle.

Supplementary Figure S3. Amounts of newly acquired ^{13}C and ^{15}N in fungal biomass per plant and in relation to the C/N ratio of the root tip.

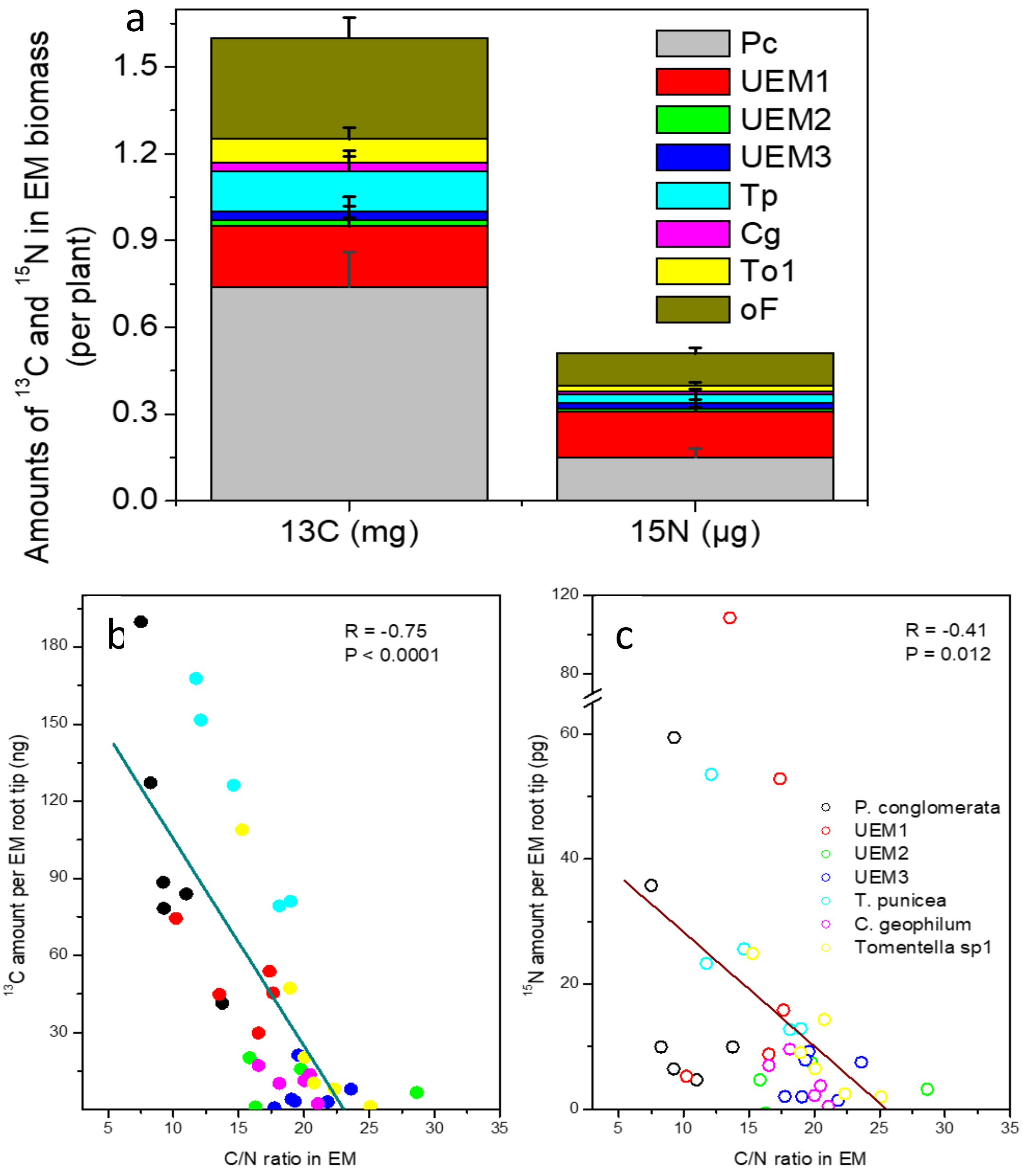

(a) Contribution of ectomycorrhizas formed with different fungal species to the pool of newly acquired ^{13}C and ^{15}N; Pc: *Pachyphlodes conglomerata* (n = 6), UEM1: uncultured EM 1 (n = 5), UEM2: uncultured EM 2 (n = 5), UEM3: uncultured EM 3 (n = 6), Tp: *Tomentella punicea* (n = 5), Cg: *Cenococcum geophilum* (n = 7), To1: *Tomentella* sp1 (n = 6). oF = other fungi (n = 8), whose individual abundances were insufficient for analyses. The contribution of oF to the pool of newly acquired ^{13}C and ^{15}N was estimated using the mean ^{13}C respective ^{15}N enrichment of mixed samples and the mean biomass of fungi reported in Table S1. **(b)** Amounts of ^{13}C and **(c)** of ^{15}N in the fungal biomass per root tip colonized with a distinct fungal species in relation to the C/N ratio of the root tip.

Supplementary Figure S4. SIMS (Secondary Ion Mass Spectrometry) imaging of ^{14}N and ^{15}N in labelled and non-labelled ectomycorrhizas with the fungus *Pachyphlodes conglomerata*

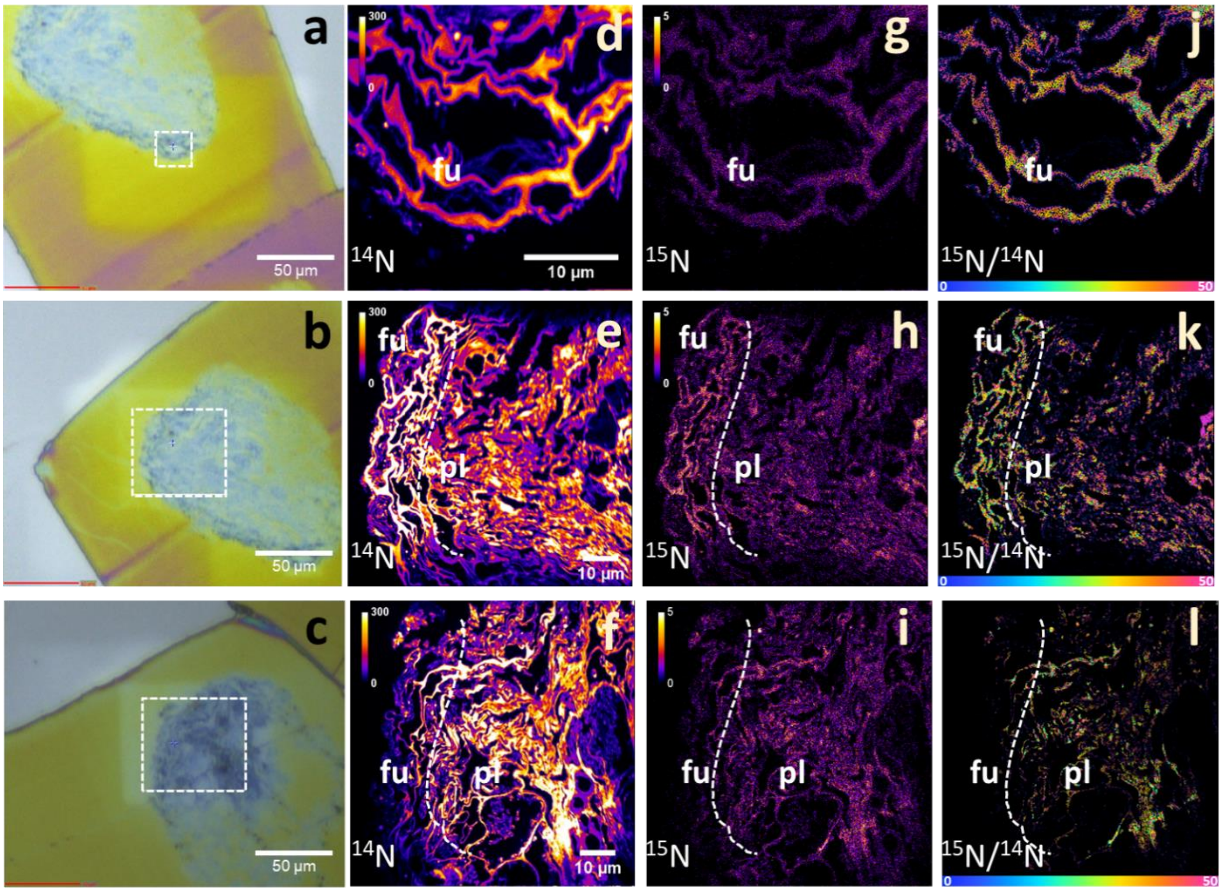

(a - c) CCD (charge-coupled device) images indicating the position for SIMS imaging. Images in the first and second row are from the same cross section, a ^{15}N labelling experiment, but at different positions and raster sizes (first row: 30 µm x 30 µm and second row: 80 µm x 80 µm). The third row of images (area: 80 µm x 80 µm) are from a cross section of a non-labelled control ectomycorrhiza with the fungus *Pachyphlodes conglomerata*. **(d - f)** SIMS images of ^{14}N in cross sections from labelled (d,e) and non-labelled controls (f). **(g - i)** SIMS images of ^{15}N in cross sections from labelled (g,h) and non-labelled controls (i). **(j - l)** SIMS images of the $^{15}N/^{14}N$ ratio in cross sections from labelled (j,k) and non-labelled controls (l). In the first row, the images display a fragment of the fungal mantle. In all other pictures, the dotted line indicates the localisation of fungal tissue (fu) and plant root cells (pl).

Supplementary Figure S5. Relationships between ^{13}C enrichment and ^{15}N enrichment with the C and the N concentrations in different EM fungal species.

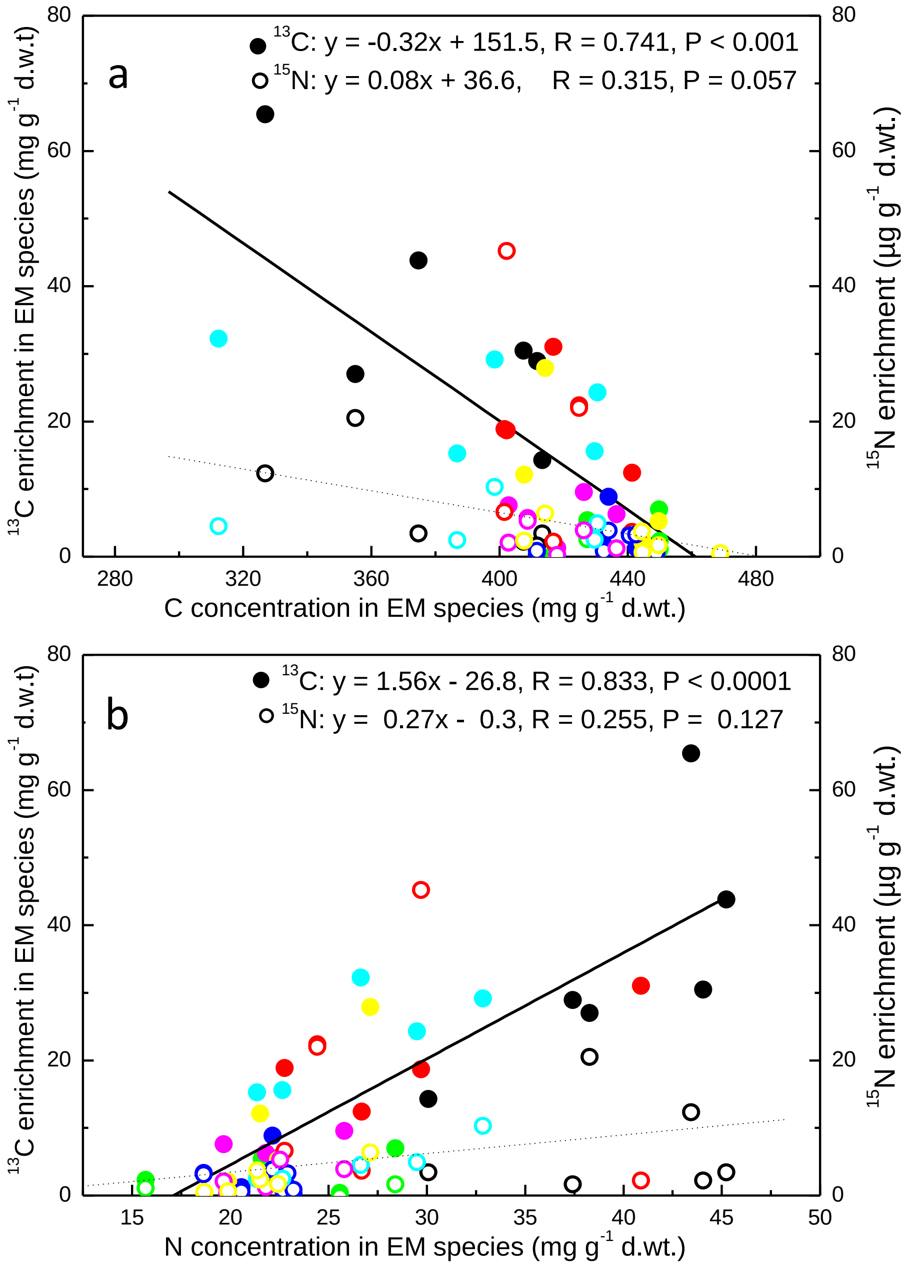

Relationship between ^{13}C enrichment (closed circles) and ^{15}N enrichment (open circles) with the C concentration **(a)** and the N concentration **(b)** in different EM fungal species. Each point is an individual measurement. Color code refers to black: *Pachyphlodes conglomerata*, red: uncultured EM 1, green: uncultured EM 2, blue: uncultured EM 3, light blue: *Tomentella punicea*, purple: *Cenococcum geophilum*, yellow: *Tomentella* sp1.

Supplementary References

[1]Pena, R., Tejedor, J., Zeller, B., Dannenmann, M. & Polle, A. Interspecific temporal and spatial differences in the acquisition of litter-derived nitrogen by ectomycorrhizal fungal assemblages. *New Phytol.* **199**, 520–528 (2013).

[2]Khokon, A. M., Janz, D. & Polle, A. Ectomycorrhizal diversity, taxon-specific traits and root N uptake in temperate beech forests. *New Phytol.* **239**, 739–751 (2023).
